# Supplementary material for: Weighted persistent homology for biomolecular data analysis
Source: Sci Rep. 2020 Feb 7;10:2079. doi: 10.1038/s41598-019-55660-3 (PMC7005716; doi:10.1038/s41598-019-55660-3)
Supplement: Supplementary file 1 — Supplementary Information. [file 41598_2019_55660_MOESM1_ESM.pdf]

Supplementary Material for
"Weighted persistent homology for biomolecular data analysis"

Zhenyu Meng[1], D Vijay Anand[1], Yunpeng Lu[2], Jie Wu[3], and Kelin Xia[1,4,*]

[1]*Division of Mathematical Sciences, School of Physical and Mathematical Sciences, Nanyang Technological University, Singapore 637371*

[2]*Division of Chemistry and Biological Chemistry, School of Physical and Mathematical Sciences, Nanyang Technological University, Singapore 637371*

[3]*School of Mathematical Sciences, Hebei Normal University, Shijiazhuang, Hebei, China 050024*

[4]*School of Biological Sciences, Nanyang Technological University, Singapore 637371*

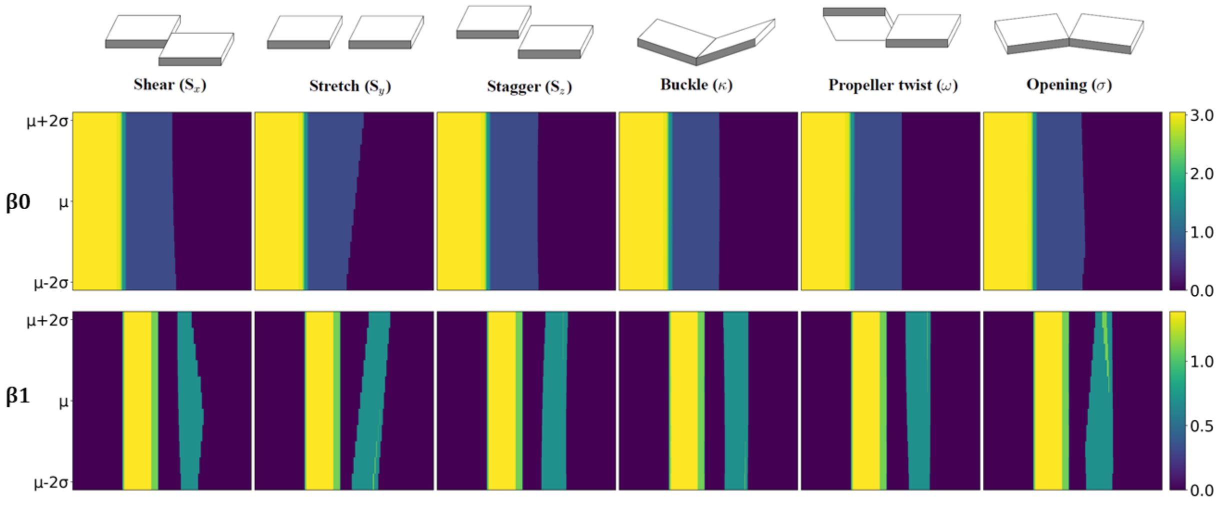

FIG. 9: The LPH based PBN image representation for each one-base-step (AT) at different helical parameter values. In the i-th PBN image, we systematically change the i-th helical parameter value from $\mu_i - 2\sigma_i$ to $\mu_i + 2\sigma_i$, with all other helical parameters remain as constants, to deliver a series of base-step structures. PBN can be calculated for each base-step structure and all of them stacked together to form a two-dimensional image. It can be seen that, both β_0 (upper figures) and β_1 (low figures) PBN functions vary with the change of helical parameter value. Note that the color values are logarithm of PBNs.

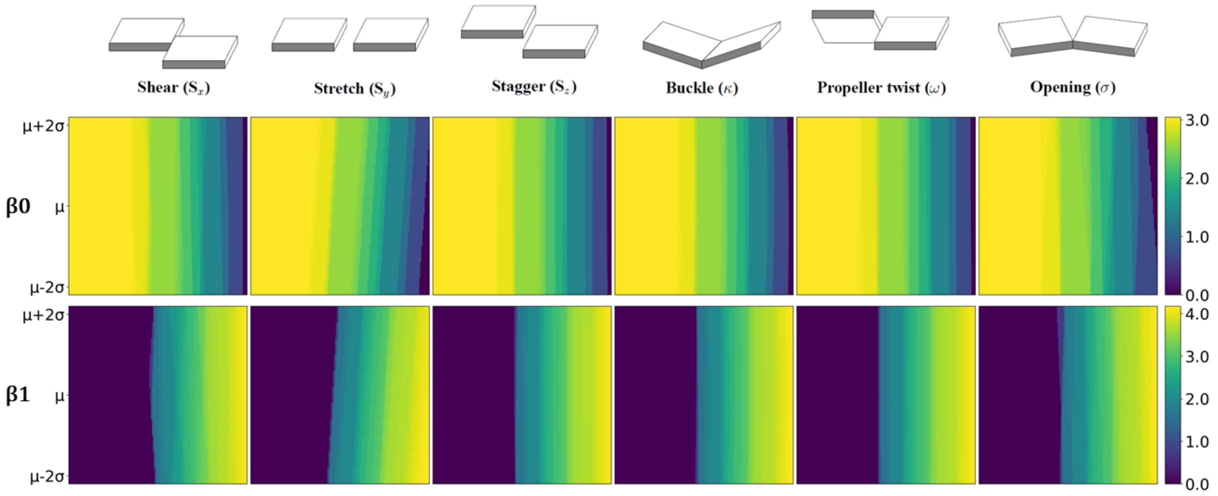

FIG. 10: The LWPH based PBN image representation for each one-base-step (AT) at different helical parameter values. In the i-th PBN image, we systematically change the i-th helical parameter value from $\mu_i - 2\sigma_i$ to $\mu_i + 2\sigma_i$, with all other helical parameters remain as constants, to deliver a series of base-step structures. PBN can be calculated for each base-step structure and all of them stacked together to form a two-dimensional image. It can be seen that, both β_0 (upper figures) and β_1 (low figures) PBN functions vary with the change of helical parameter value.

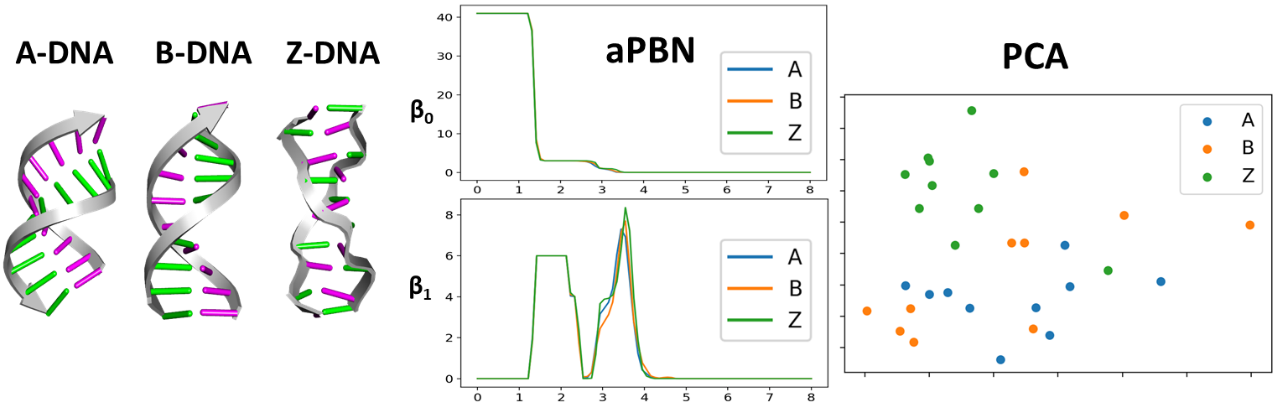

FIG. 11: LPH based classification of three DNA types, i.e., A-DNA, B-DNA, and Z-DNA. The average persistent Betti number (aPBN) from our LPH for three types of DNAs. We discretize the aPBN equally into a series of numbers and use these values as features for PCA. It can be seen that LPH based aPBN and PCA results cannot discriminate three DNA types.

TABLE II: The A-, B- and Z-types of proteins used in our paper. For each type, we arbitrary choose 10 PDB structures from the PDB databank.

A	B	Z
1DNZ	1BNA	1ICK
1QPH	1D29	1WOE
2D47	1D65	1XAM
2D94	2L8Q	3P4J
3V9D	2MCI	3WBO
440D	3IXN	4HIG
4IZQ	3U2N	4OCB
5MVT	4C64	5JZQ
5WV7	5T4W	6AQV
5XK0	6CQ3	6BST

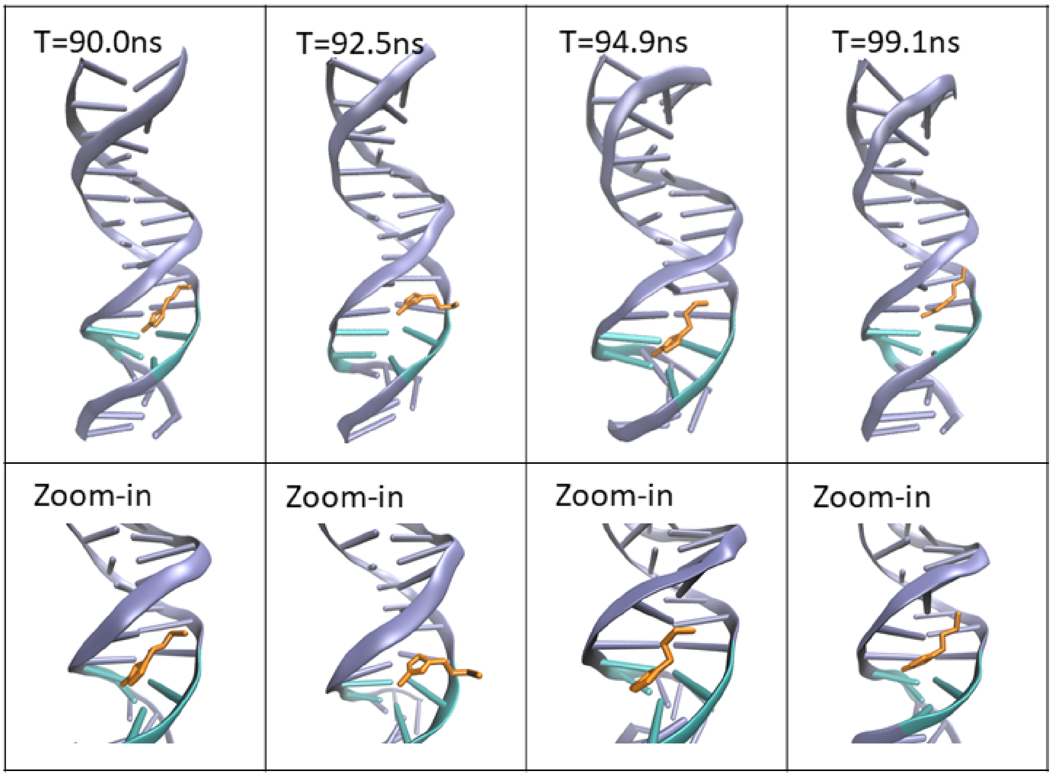

FIG. 12: The illustration of ion-DNA binding in different meta-states. Four different configurations are extracted from four different regions in our LWPH model (as in Figure 14), respectively. It can be seen that $BMIM^+$ cation stays in minor grove at beginning, i.e., t=90.0 ns, dissociates at t=92.5 ns, rebinds to DNA at t=94.9 ns, and shifts upward at the end of the simulation, i.e., t=99.1 ns.

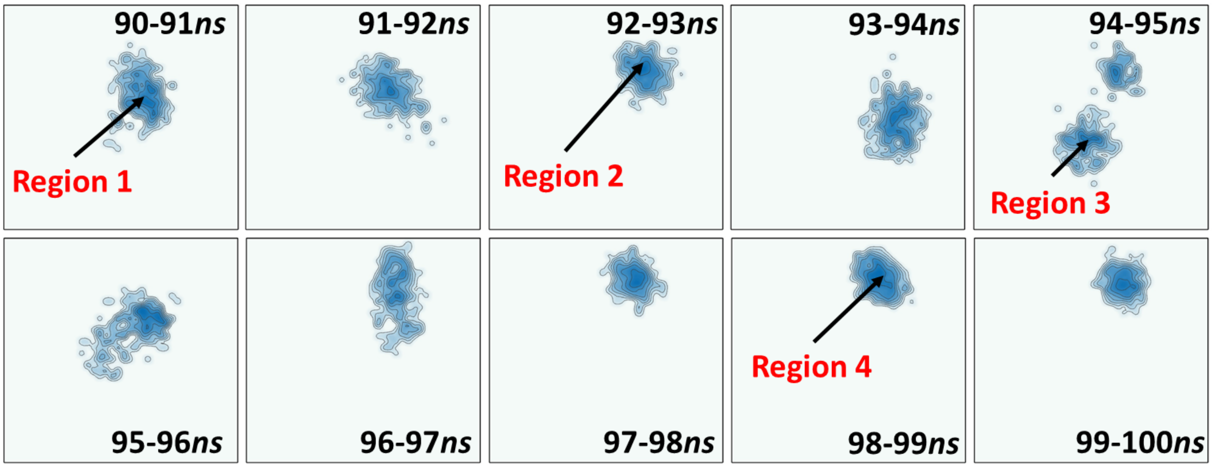

FIG. 13: The IL1 contour map generated from helical parameter based PCA models. Only the trajectories during the simulation time 90 to 100 nanoseconds are considered. Four different local regions can be identified, meaning that there are four different DNA configuration states.

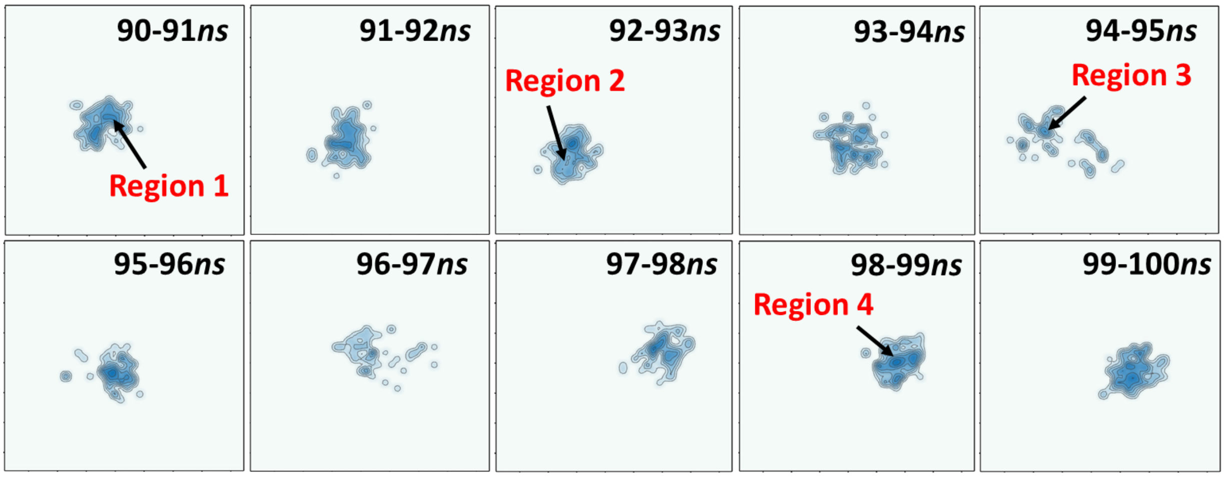

FIG. 14: The IL1 contour map generated from our LWPH based PCA models. Only the trajectories during the simulation time 90 to 100 nanoseconds are considered. Four different local regions can be identified, meaning that there are four different DNA configuration states. More importantly, these four states and the transition between the four states are highly consistent with the prediction from the helical parameter based models as in Figure 13.

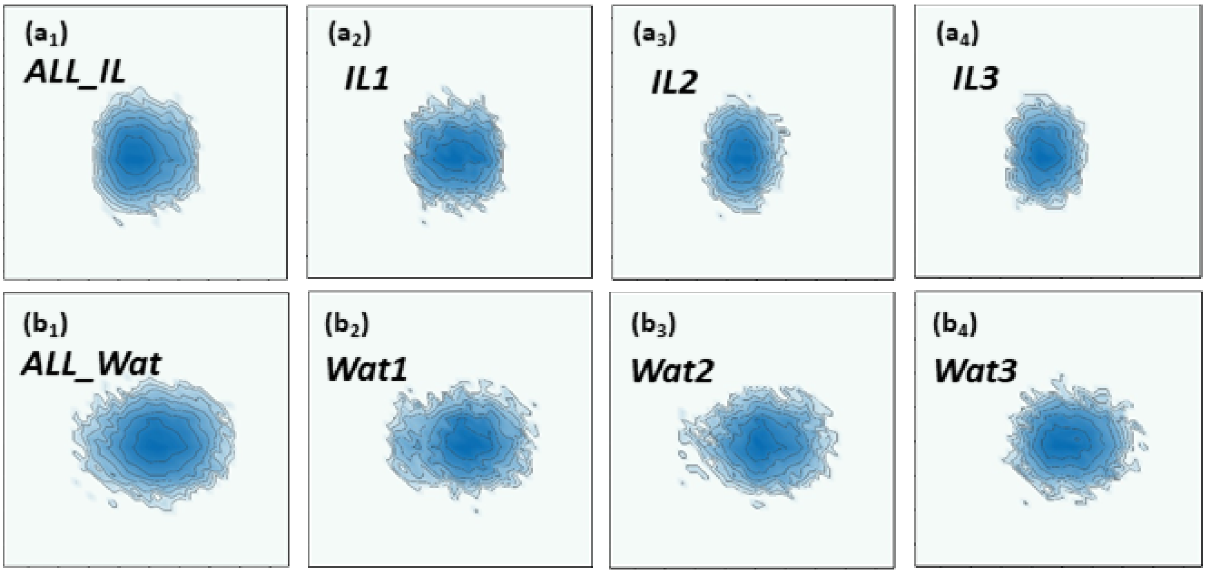

FIG. 15: The contour map generated from atom-coordinate PCA models from DNA configurations in IL and WAT environments. Similar to Figures (7) and (8), the x-axis and y-axis are the first and second principal components. Same notations for ($\mathbf{a}_1$) to ($\mathbf{a}_4$) and ($\mathbf{b}_1$) to ($\mathbf{b}_4$) are used. The confinement effect can be observed. However, the two center distribution of DNA configurations in IL environment are not observed.

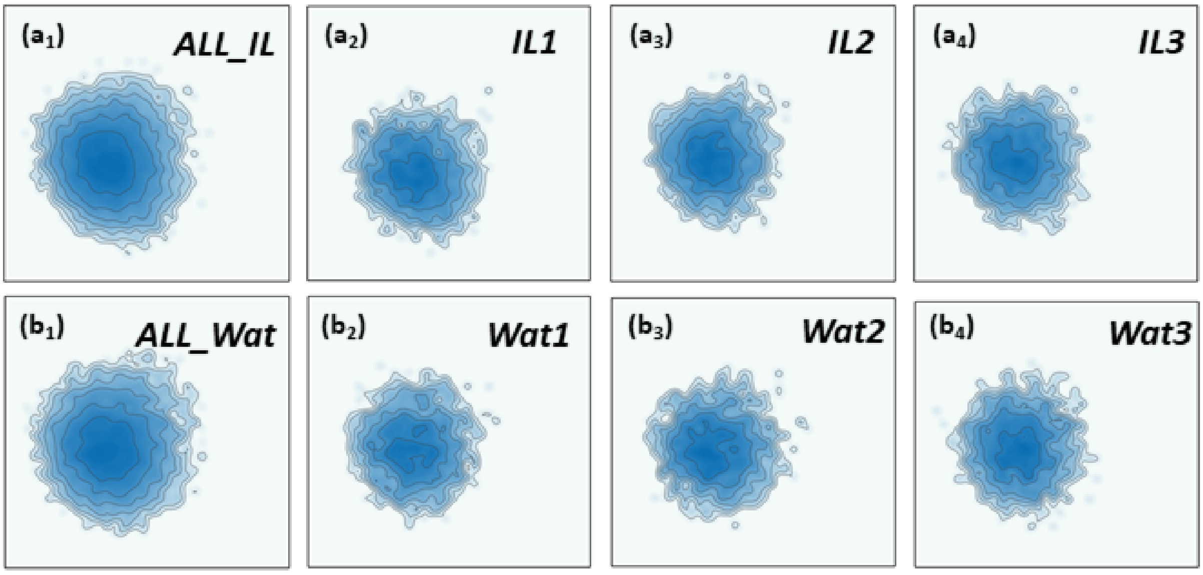

FIG. 16: The contour map generated from LPH based PCA models from DNA configurations in IL and WAT environments. Similar to Figures (7) and (8), the x-axis and y-axis are the first and second principal components. Same notations for ($\mathbf{a}_1$) to ($\mathbf{a}_4$) and ($\mathbf{b}_1$) to ($\mathbf{b}_4$) are used. However, confinement effect and two center distribution of DNA configurations in IL environment are not observed.

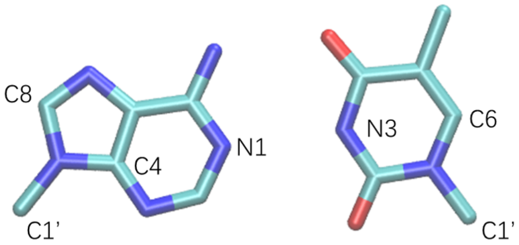

FIG. 17: The selected atoms in the CEHS scheme. Note that only $C8$, $C4$, $N1$ and $C1'$ of purine base and $N3$, $C6$ and $C1'$ of pyrimidine base is considered in CEHS model.

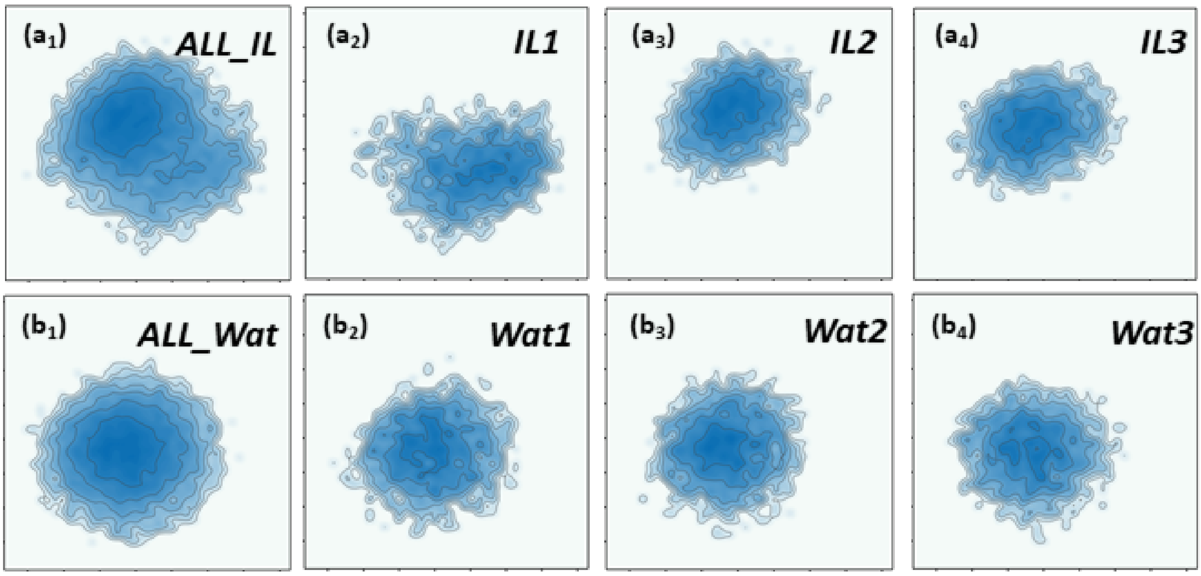

FIG. 18: The contour map generated from our LWPH based PCA models for selected atoms (as in Figure 17) from DNA configurations in IL and WAT environments. Similar to Figures (7) and (8), the x-axis and y-axis are the first and second principal components. Same notations for ($\mathbf{a}_1$) to ($\mathbf{a}_4$) and ($\mathbf{b}_1$) to ($\mathbf{b}_4$) are used. Further, the same confinement effect and two center distribution of DNA configurations in IL environment as in Figures (7) and (8) are observed.

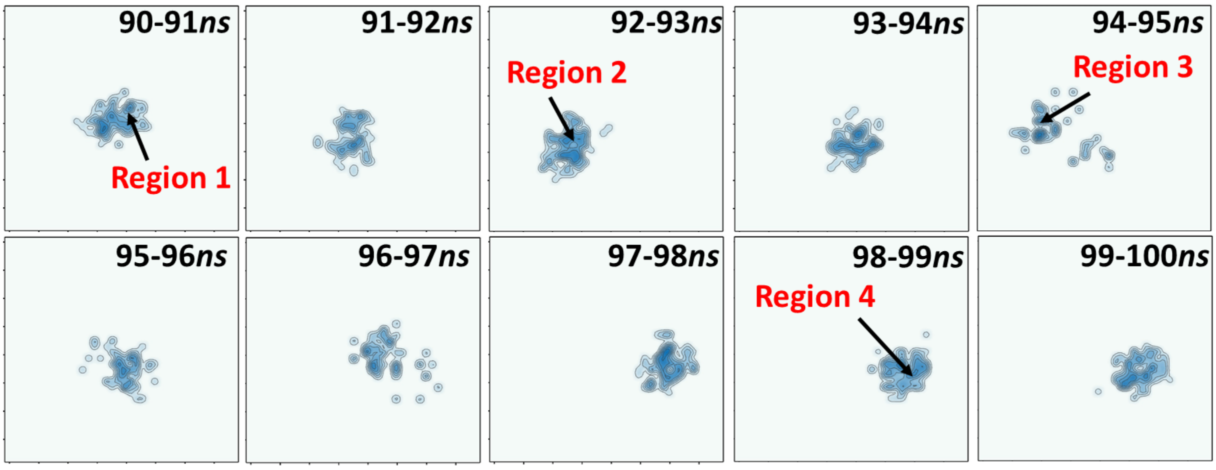

FIG. 19: The IL1 contour map generated from PCA of our selected atom (as in Figure (17)) based LWPH. Only the trajectories during the simulation time 90 to 100 nanoseconds are considered. Four different local regions can be identified, meaning that there are four different DNA configuration states. More importantly, these four states and the transition between the four states are highly consistent with the prediction from the helical parameter based models as in Figures (13) and (14).
